# Can DCE-MRI Explain the Heterogeneity in Radiopeptide Uptake Imaged by SPECT in a Pancreatic Neuroendocrine Tumor Model?

**DOI:** 10.1371/journal.pone.0077076

**Published:** 2013-10-08

**Authors:** Karin Bol, Joost C. Haeck, Harald C. Groen, Wiro J. Niessen, Monique R. Bernsen, Marion de Jong, Jifke F. Veenland

**Affiliations:** 1 Biomedical Imaging Group Rotterdam, Departments of Radiology and Medical Informatics, Erasmus Medical Center, Rotterdam, The Netherlands; 2 Department of Radiology, Erasmus Medical Center, Rotterdam, The Netherlands; 3 Department of Nuclear Medicine, Erasmus Medical Center, Rotterdam, The Netherlands; 4 Imaging Science and Technology, Faculty of Applied Sciences, Delft University of Technology, Delft, The Netherlands; Genentech, United States of America

## Abstract

Although efficient delivery and distribution of treatment agents over the whole tumor is essential for successful tumor treatment, the distribution of most of these agents cannot be visualized. However, with single-photon emission computed tomography (SPECT), both delivery and uptake of radiolabeled peptides can be visualized in a neuroendocrine tumor model overexpressing somatostatin receptors. A heterogeneous peptide uptake is often observed in these tumors. We hypothesized that peptide distribution in the tumor is spatially related to tumor perfusion, vessel density and permeability, as imaged and quantified by DCE-MRI in a neuroendocrine tumor model. Four subcutaneous CA20948 tumor-bearing Lewis rats were injected with the somatostatin-analog ^111^In-DTPA-Octreotide (50 MBq). SPECT-CT and MRI scans were acquired and MRI was spatially registered to SPECT-CT. DCE-MRI was analyzed using semi-quantitative and quantitative methods. Correlation between SPECT and DCE-MRI was investigated with 1) Spearman’s rank correlation coefficient; 2) SPECT uptake values grouped into deciles with corresponding median DCE-MRI parametric values and vice versa; and 3) linear regression analysis for median parameter values in combined datasets. In all tumors, areas with low peptide uptake correlated with low perfusion/density/ /permeability for all DCE-MRI-derived parameters. Combining all datasets, highest linear regression was found between peptide uptake and semi-quantitative parameters (R^2^>0.7). The average correlation coefficient between SPECT and DCE-MRI-derived parameters ranged from 0.52-0.56 (p<0.05) for parameters primarily associated with exchange between blood and extracellular extravascular space. For these parameters a linear relation with peptide uptake was observed. In conclusion, the ‘exchange-related’ DCE-MRI-derived parameters seemed to predict peptide uptake better than the ‘contrast amount- related’ parameters. Consequently, fast and efficient diffusion through the vessel wall into tissue is an important factor for peptide delivery. DCE-MRI helps to elucidate the relation between vascular characteristics, peptide delivery and treatment efficacy, and may form a basis to predict targeting efficiency.

## Introduction

The goal of tumor treatment is to efficiently deliver and distribute the treatment agent over the whole tumor to maximize treatment efficacy. In certain parts of the tumor, however, if the treatment agent concentration is too low these parts may escape treatment. Whereas the distribution of most treatment agents cannot be imaged, the delivery and uptake of radiolabeled peptides (which bind to receptors on the tumor cell membrane) can be visualized using single-photon emission computed tomography (SPECT). Neuroendocrine tumors overexpressing somatostatin receptors on the cell membrane can be imaged with SPECT using radiolabeled somatostatin-derived peptide analogs that target these receptors [1,2,3,4].

**Figure 1:**
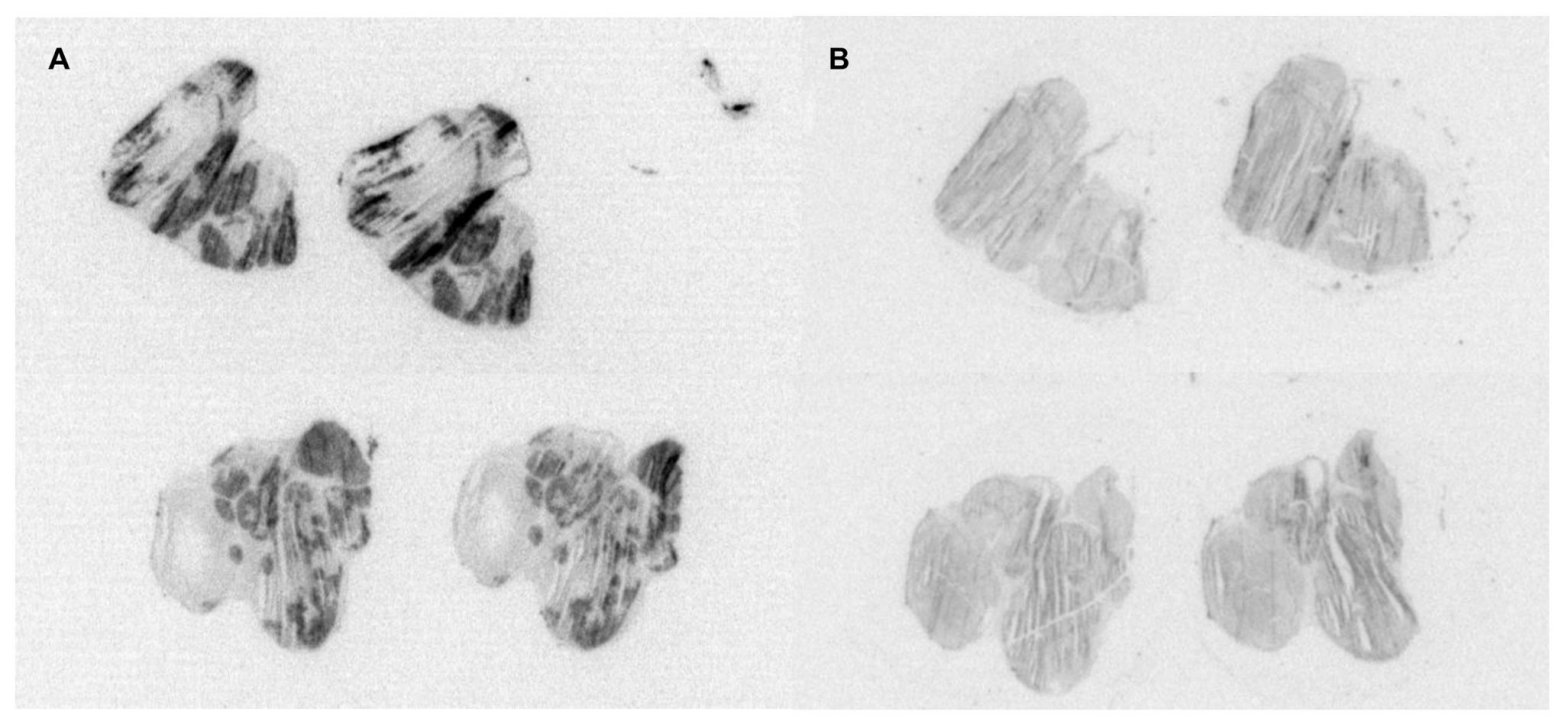
Heterogeneous ex vivo peptide uptake versus homogeneous receptor expression. Illustration of the heterogeneous SPECT peptide uptake in ex vivo autoradiography (A), with the corresponding homogeneous receptor expression in in vitro autoradiography (B), for a pancreatic neuroendocrine tumor model (CA20948).

Using these radiolabeled somatostatin-derived peptide-analogs, neuroendocrine tumors can also be treated by local irradiation using peptide receptor radionuclide therapy (PRRT) [5,6,7,8,9]. The current prognosis for patients with pancreatic neuroendocrine tumors is poor when the tumor has metastasized [7,10,11]. In the case of metastatic spread, surgical treatment is often not possible [7,12] and other treatment options are limited. Therapy using radiolabeled somatostatin analogs is an important novel therapeutic option in patients with irresectable or metastasized pancreatic neuroendocrine tumors: a benefit of overall survival of several years has been reported [7,13,14,15]. However, a pre-clinical study [16] revealed heterogeneous radioactivity distribution in the tumor in ex vivo autoradiography, even though in vitro autoradiography demonstrated homogeneous somatostatin-receptor distribution in vital tumor regions, as illustrated in Figure 1. This heterogeneous peptide uptake and distribution in vivo may hamper effective treatment with PRRT.

The heterogeneous peptide distribution may be governed by physiological factors, including vascular tumor characteristics. These characteristics can be studied non-invasively with dynamic contrast-enhanced magnetic resonance imaging (DCE-MRI), which allows to visualize and quantify the concentration of contrast agent in blood vessels and leakage of this contrast into tissues. With these images, the extent of perfusion/density/permeability of the vessels of the tumor can be examined and spatially correlated to the heterogeneous uptake of radiolabeled peptide observed with SPECT. This correlation can provide insight into the influence of tumor perfusion, vessel density and permeability on peptide distribution, and may help to improve treatment of neuroendocrine tumors.

The aim of this study was to assess the spatial correlation of tumor perfusion, vessel density and permeability with the uptake and distribution of radiolabeled peptides in a pancreatic neuroendocrine tumor model (CA20948) in rats.

## Material and Methods

### Animal and tumor model

For this study male Lewis rats (n=4, Harlan-CPB, Austerlitz, the Netherlands), weighing ± 300 g were used. All investigations were carried out in accordance with the requirements of the institutions concerned and conformed with the requirements in the Netherlands regarding animal studies. The protocol was approved by the Ethics Committee for Animal Research at Erasmus MC, Rotterdam in accordance with Dutch law (DEC no. EMC 2042).

During imaging the animals were anesthetized with isoflurane (Nicholas Pyramal Ltd., London, UK), body temperature was controlled using a heated bed, and all efforts were made to minimize suffering. Pancreatic tumor cells (10^6^ CA20948 cells) were inoculated subcutaneously in the right flank. When the tumors reached a diameter of at least 1.5 cm the animals were imaged. Animals were placed in a dedicated holder to ensure the same position of the animal and tumor during both SPECT-CT and MRI imaging. After completion of the examinations the animals were sacrificed.

### Radiolabeling

Radiolabeling of ^111^In-DTPA-octreotide (OctreoScan, Covidien, Petten, The Netherlands) was performed by a 30-min incubation of pentetreoide and ^111^InCl_3_. A specific radioactivity of 7.1 MBq per nmol octreotide (labeling efficiency of >98%) was used as this could be prepared every day of the week, thereby ensuring that all animals received the same amount of radioactivity and peptide mass.

### SPECT-CT imaging

One hour prior to SPECT imaging, all animals received 200 µl 50 ± 2 MBq OctreoScan (1408.5541 g/mol) through the tail vein under isoflurane anesthesia. SPECT-CT images were acquired using a 4-head multi-pinhole NanoSPECT/CT camera (Bioscan, Mediso Medical Imaging Systems, Hungary). The energy peak settings were 171 and 245 keV (window width ± 10%) for ^111^In. Nine-pinhole apertures (diameter 2.5 mm) were used on each camera head, with a transaxial field of view (FOV) of 60 mm. SPECT images were acquired using 36 projections, 120 s/projection, quality factor 1, and a total scan time of 36 min. SPECT images were reconstructed using the OSEM method, with 9 iterations and a voxel size of 0.5x0.5x0.5 mm. CT images were acquired using the following settings: 360 projections, 45kVp tube voltage, 1000 ms exposure time, a scan time of 9 min, and a voxel size of 0.2x0.2x0.2 mm. In a post-processing step, the SPECT images were resampled to the CT resolution. The counts in the SPECT images were converted to kBq/voxel by the scanner software; these values were used for the spatial correlation. All data will be made available upon request.

### MRI imaging

Directly after SPECT-CT imaging, MRI images were acquired. Imaging was performed on a 7.0 Tesla dedicated animal scanner (Discovery MR901, Agilent Technologies/GE Healthcare) using a 4-channel surface receiver coil (Rapid MR International, Ohio, USA). Prior to the DCE-MRI sequence two additional scans were acquired: 1) high resolution susceptibility-weighted images (SWI) for registration purposes using a gradient echo sequence (repetition time (TR) 10 ms; echo time (TE) 4.5 ms; 6° flip angle; FOV 4 cm; voxel size 0.098x0.098x0.30 mm); and 2) T_1_-weighted images with varying TRs for calibration purposes (TR 200, 400, 800, 1200, 2400 ms; TE 8.0 ms; 90° flip angle; FOV 5.0 cm; voxel size 0.207x0.207x0.80 mm).

For accurate DCE-MRI quantification, temporal resolutions < 5 s are advised for animal models to ensure reliable model fitting and parameter estimation [17]. Therefore, DCE-MRI images were acquired using time-resolved imaging of contrast kinetics (TRICKS) to achieve a high temporal resolution: 96 images were made with a temporal resolution of 4.3 s. These images were acquired in two sequential acquisitions with an interval of 60 s between the acquisitions, with a total time span of 8 min. Other acquisition parameters for TRICKS were: TR 3.4 ms, TE 1.0 ms, 10° flip angle, FOV 5.0 cm, and voxel size 0.195x0.195x0.50 mm. The contrast agent (Gadovist, Bayer, Mijdrecht, The Netherlands) used during MRI scanning was administered intravenously in the tail vein. A single dosage of 0.2 mmol/kg Gadovist was injected at a constant speed of 1.2 mmol/kg*min. All data will be made available upon request.

### Data analysis

#### Registration MRI with SPECT-CT

To enable spatial correlation between MRI and SPECT images, registration between both modalities is required. SPECT and CT images were automatically aligned by dedicated software in the SPECT-CT scanner (InVivo Scope software, Bioscan). For an unbiased registration between DCE-MRI and SPECT-CT a separate MRI sequence is required; otherwise, direct use of DCE-MRI for registration purposes could result in overestimation of the correlation between DCE-MRI and SPECT-CT. Therefore, the SWI were acquired for registration purposes.

The tumors were manually delineated on the SWI and CT scans and, using these contours, tumor masks were generated. The SWI masks were registered to the CT masks using Elastix [18], subsequently applying a rigid and affine registration scheme. Since use of the dedicated animal holder ensured that no deformation occurred in tumors between scanning (MRI/SPECT-CT), non-rigid registration was not necessary. The transformation parameters acquired from both registration schemes were then used to transform the DCE-MRI images to the CT images.

#### DCE-MRI quantification

To derive features from the DCE-MRI data, both a semi-quantitative and a quantitative analysis method were applied. Semi-quantitative methods can be computed without acquiring a T1-weighted sequence of scans for calculating the T10-map, and are therefore better suited for clinical applications. Analyses were performed using in-house developed software based on MATLAB (MathWorks, Natick, MA, USA).

#### Semi-quantitative analysis

Signal enhancement over time curves (SI curves) were constructed for all voxels inside the tumor mask. Six semi-quantitative parameters were calculated from the SI curve: 1) maximum enhancement (Smax), 2) time-to-peak (TTP), 3) area under the whole curve (AUC), 4) area under the curve for the first 60 s (AUC 60), 5) wash-in, and 6) wash-out. The wash-in was calculated as the slope between the 10% point and the 90% point on the enhancement curve, and the wash-out as the slope of the SI curve between the point of maximum enhancement and the last time point, according to Parker et al. [19]. Since the signal intensity in MRI is relative and can differ between subjects and scanners, semi-quantitative parameters were normalized to enable comparison of these parameters between animals. Each parameter was linearly scaled between the minimum and maximum by using x_norm_=(x -x_min_)_/_(x_max_-x_min_) resulting in values between 0 and 1.

#### Quantitative analysis

The standard compartment model of Tofts et al. [20] was used to model the pharmacokinetic behavior of contrast agent inside the tumor. From this model the following parameters were calculated: K^trans^ (s^-1^), the capillary transfer constant between the intravascular and the extravascular extracellular compartments, and k_ep_ (s^-1^), the contrast exchange rate from the extravascular extracellular space to the intravascular compartment. Physiologically, K^trans^ is a combination of blood flow and capillary permeability inside the tumor and its exact physiological interpretation depends on the balance between this perfusion and permeability [20].

To calculate quantitative DCE-MRI parameters, absolute MRI values are needed. For this purpose, T1-weighted sequences with varying repetition times were acquired for the computation of the T_1_(0)-map. Using this map, the SI curves were converted to contrast-concentration curves. The standard Tofts compartment model was fitted to the contrast-concentration curves, resulting in the parametric maps for K^trans^ and k_ep_. For this method an arterial input function (AIF) was required, which was determined per dataset by fitting a bi-exponential equation [21] to an average contrast-concentration curve acquired from the femoral artery.

#### Correlating DCE-MRI-derived parameters with SPECT peptide uptake

For correlation of the DCE-MRI-derived parameters with SPECT uptake, only voxels within the tumor boundary were taken into account, excluding the tumor feeding vessels; all parameters are computed over the same set of voxels. However, as shown by others [22], the standard compartment Tofts model sometimes poorly fits for voxels with low contrast enhancement. In these instances K^trans^ and k_ep_ have very high, non-physiological values. For these voxels (with no or very little contrast agent accumulation in the DCE-MRI images) K^trans^ and k_ep_ were set to zero.

The correlation between SPECT and DCE-MRI was investigated using three different methods. First, Spearman’s rank correlation coefficients between DCE-MRI-derived parameters and SPECT-peptide uptake were calculated per dataset over all tumor voxels. Secondly, for each dataset, the range of SPECT values was sorted and divided into deciles. For each subset of voxels within the SPECT decile region, the same region in the DCE-MRI parameter map was located, and the median DCE-MRI parameter values for that decile region were calculated. Similarly, for each dataset the range of DCE-MRI parameter values was sorted and divided into deciles, and for each DCE decile region the corresponding median SPECT values were calculated. Thirdly, median DCE-MRI parameter values for all 4 datasets were grouped based on the SPECT deciles and a linear regression analysis was performed.

## Results

**Figure 2:**
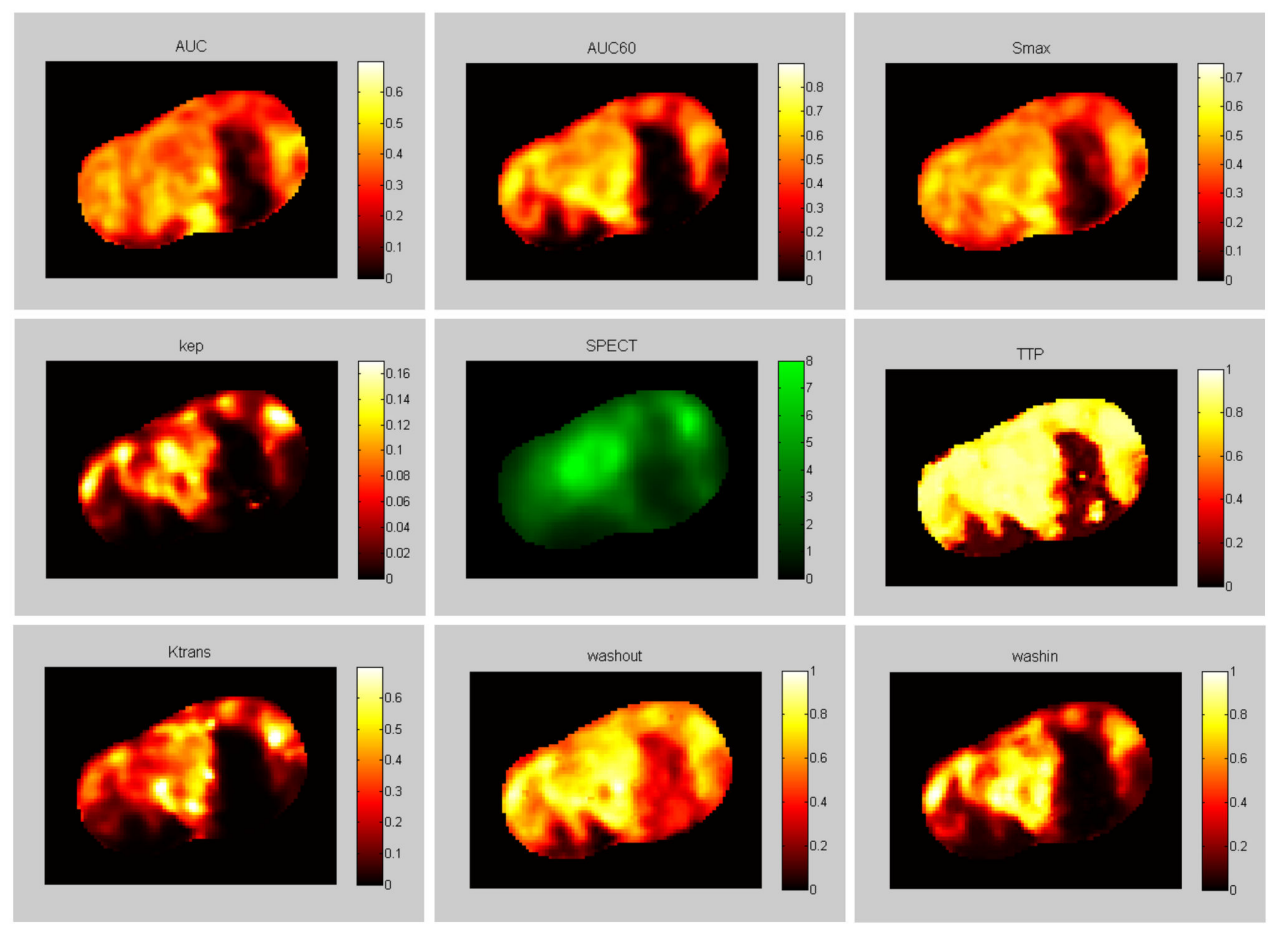
Visualization of the correspondence between SPECT-peptide uptake and DCE-MRI-derived parameters. Color coded images of SPECT peptide uptake (in Bq) (green) and different DCE-MRI parameters (red) for one slice through the tumor. Clockwise starting from the top left: AUC, area under the whole curve; AUC60, area under the curve for the first 60 s; Smax, maximum enhancement; TTP, time to peak; wash-in, slope between the 10% point and the 90% point on the enhancement curve [19]; wash-out [19]; K^trans^ (s^-1^) capillary transfer constant [20]; k_ep_ (s^-1^), contrast exchange rate [20]. All semi-quantitative parameters were normalized and TTP values were inverted for visualization purposes.

Figure 2 shows the overlap between SPECT peptide uptake (in green) and the different DCE-MRI parameters (in red); it can be seen that low DCE-MRI parameter values coincide with low SPECT values. However, high DCE-MRI parameter values do not always result in high SPECT uptake, as can be seen in the leftmost part of the tumor. Only a few voxels, in the middle of the dark part, were set to zero due to non-physiological values in the K^trans^ and k_ep_ maps. For visualization purposes, the TTP values were inverted.

The spatial correlation between the SPECT peptide uptake and the different DCE-MRI parameters was computed using Spearman’s rank correlation coefficient (ρ) (Table 1). The average ρ was ≥ 0.52 for all parameters, except for AUC and Smax. For the individual tumors the highest ρ (0.61-0.63) was found for wash-in, wash-out, TTP, K^trans^, and k_ep_.

**Table 1:** Mean and standard deviation (SD) of Spearman’s correlation coefficient (ρ) between SPECT peptide uptake and different DCE-MRI parameters, calculated over all datasets.

**DCE-MRI parameter**	**Mean ρ (range)**	**SD**
AUC	0.38 (0.27-0.56)	0.13
AUC60	0.52 (0.43-0.56)	0.06
Smax	0.40 (0.25-0.55)	0.12
TTP	0.55 (0.45-0.62)	0.09
wash-in	0.56 (0.47-0.61)	0.06
wash-out	0.55 (0.45-0.61)	0.09
K^trans^	0.55 (0.45-0.61)	0.07
k_ep_	0.55 (0.46-0.63)	0.08

AUC, area under the whole curve

AUC60, area under the curve for the first 60 s

Smax, maximum enhancement

TTP, time to peak wash-in, slope between the 10% point and the 90% point on the enhancement curve [19]

wash-out, slope between the point of maximum enhancement and the last time point [19];

K^trans^ (s^-1^), capillary transfer constant [20]

k_ep_ (s^-1^), contrast exchange rate [20]

**Figure 3:**
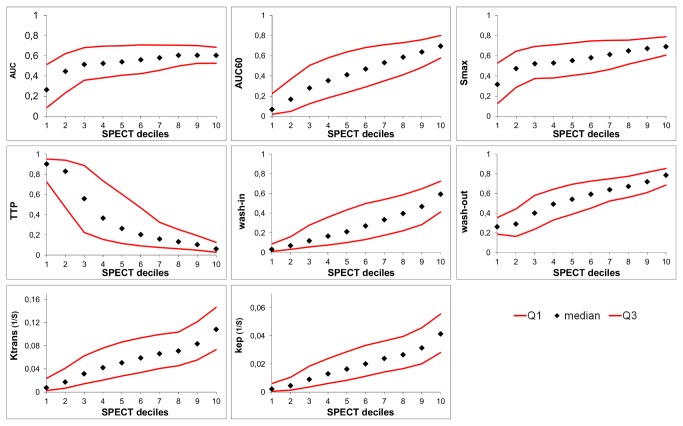
SPECT decile groups with corresponding median DCE-MRI parameter values. The median, Q1, and Q3 DCE-MRI parameter values for the different SPECT decile bins for one tumor. AUC, area under the whole curve; AUC60, area under the curve for the first 60 s; Smax, maximum enhancement; TTP, time to peak; wash-in, slope between the 10% point and the 90% point on the enhancement curve [19]; wash-out, slope between the point of maximum enhancement and the last time point [20]; K^trans^ (s^-1^) capillary transfer constant [20]; k_ep_ (s^-1^), contrast exchange rate [20]. Semi-quantitative parameters were normalized.

Figure 3 shows the median DCE-MRI parameter values, together with the Q1 (first quartile) and Q3 (third quartile) values, per SPECT decile for one tumor. All DCE-MRI parameters, except for TTP, showed an increase with increasing SPECT values. Since TTP quantifies elapsed time until maximum enhancement, TTP decreased with increasing SPECT deciles. The increase for the AUC and Smax in the high SPECT deciles was less steep compared with the other DCE-MRI parameters. The other 3 datasets yielded similar results (not shown).

**Figure 4:**
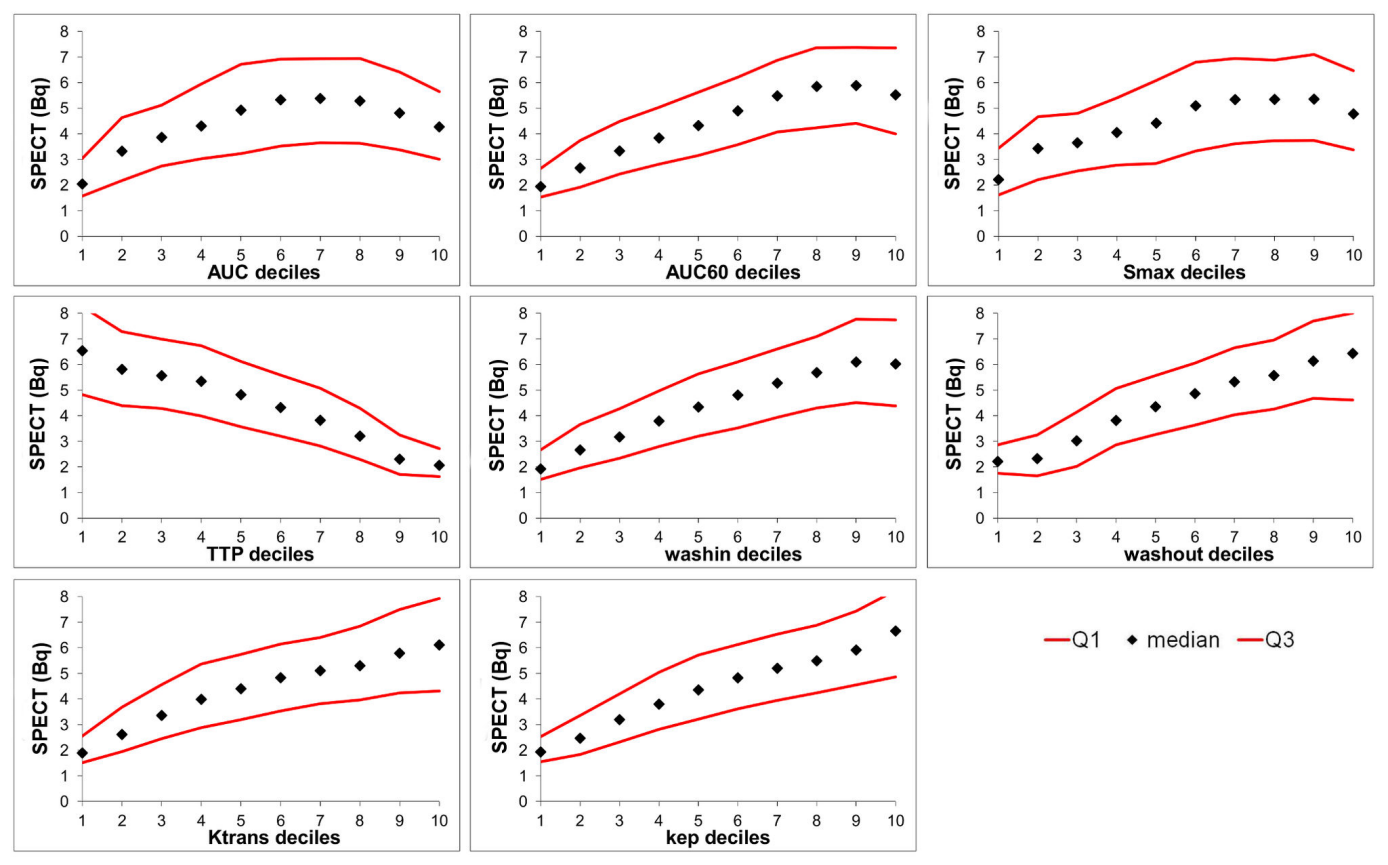
DCE-MRI parameter decile groups with corresponding median SPECT-peptide uptake values. The median, Q1, and Q3 SPECT values (Bq) for the different DCE-MRI parameter decile bins for one tumor. AUC, area under the whole curve; AUC60, area under the curve for the first 60 s; Smax, maximum enhancement; TTP, time to peak; wash-in, slope between the 10% point and the 90% point on the enhancement curve [19]; wash-out, slope between the point of maximum enhancement and the last time point [19]; K^trans^ (s^-1^) capillary transfer constant [20]; k_ep_ (s^-1^), contrast exchange rate [20].

Figure 4 shows the inverse relation between SPECT uptake and the DCE-MRI parameters for one tumor: the median SPECT values, together with the Q1 and Q3 values, are plotted as a function of the different DCE-MRI parameter deciles. For all parameters, except for AUC, AUC60 and Smax, a monotone increasing or decreasing function was observed. For AUC, AUC60 and Smax a maximum in the SPECT value is already reached for lower DCE-MRI parameter values. From that point on, higher DCE-MRI parameter values correspond to sub-maximal SPECT values. The other 3 tumors showed similar relations for all parameters (not shown).

**Figure 5:**
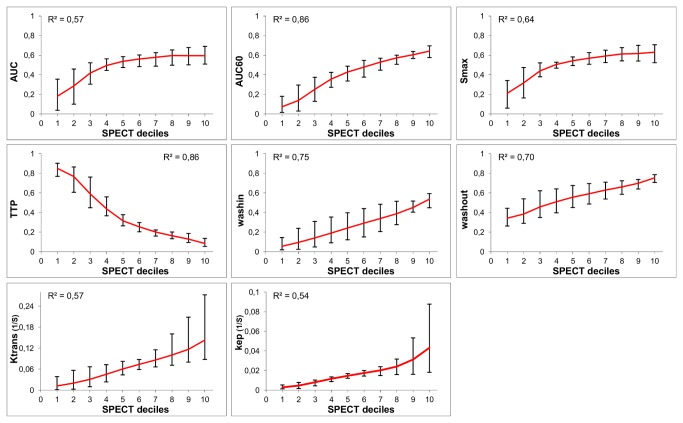
Linear regression analysis on the grouped datasets. The median value per parameter for the SPECT decile bins calculated over all datasets, together with highest and lowest median value per parameter from the 4 individual datasets, and R^2^ value per parameter from linear regression analysis (p<0.05). AUC, area under the whole curve; AUC60, area under the curve for the first 60 s; Smax, maximum enhancement; TTP, time to peak; wash-in, slope between the 10% point and the 90% point on the enhancement curve [19]; wash-out, slope between the point of maximum enhancement and the last time point [19]; K^trans^ (s^-1^) capillary transfer constant [20]; k_ep_ (s^-1^), contrast exchange rate [20]. Semi-quantitative parameters were normalized.

In Figure 5 all datasets are combined. The median value per parameter as a function of the SPECT decile values is plotted, together with the highest and lowest median parameter value of the 4 individual datasets. Linear regression analysis showed a significant (p < 0.05) correlation between the SPECT uptake and all DCE-MRI parameters. A high correlation (R^2^ > 0.7) was observed for AUC60, TTP, wash-in, and wash-out. For the other DCE-MRI parameters, the R^2^ was lower (0.55-0.65) due to the non-linear relation (AUC, Smax) or to the spread of median values between the different datasets.

## Discussion

This study investigated the influence of tumor perfusion, vessel density and permeability on the tumor uptake and distribution of radiolabeled peptides. For this, we evaluated the spatial correlation between DCE-MRI-derived perfusion parameters and the radiolabeled peptide distribution in SPECT-CT for the CA20948 somatostatin receptor-positive pancreatic tumor model. First, we spatially registered the DCE-MRI images to the SPECT-CT images, followed by analysis of DCE-MRI data using a semi-quantitative and a quantitative analysis method. Voxel-based analysis over the whole tumor showed a clear relationship between SPECT uptake values and the DCE-MRI parameters AUC60, TTP, wash-in, wash-out, K^trans^ and k_ep_. Combining all datasets showed a high linear regression correlation between peptide uptake and AUC60, TTP, wash-in, and wash-out (R^2^ > 0.7).

It is established that tumors (even within the same type and grade) can be very heterogeneous and quantitative imaging biomarkers are useful to provide important reproducible estimates of this tumor heterogeneity in vivo [23]. Tumor heterogeneity is an important factor since it affects treatment delivery and response. The efficacy of DCE-MRI-derived parameters for assessing this heterogeneity and its effect on therapy response is therefore an interesting topic of research [24,25,26]. However, in such studies anti-tumor response is the main treatment outcome, and no insight is obtained on the spatial correlation between the DCE-MRI parameter values and drug delivery. SPECT enables imaging of the delivery of the peptide and direct spatial correlation of the distribution of the peptide to the DCE-MRI parametric maps.

One study reported a visual correlation between gadolinium enhancement in MRI images and ^111^In-pentetreotide (OctreoScan) SPECT images in brain tumors [27]. In a study by Miyazaki et al. [12], DCE-MRI-derived parameters were used to monitor changes in patients with neuroendocrine liver metastases treated using yttrium 90 (90Y)-labeled octreotide (90Y-DOTATOC) and to predict treatment response. However, in that study no SPECT images were acquired for a voxel-wise correlation of the peptide distribution and DCE-MRI parametric maps. In two recent PET studies, a spatial correlation of parameters derived from DCE-CT or DCE-MRI with the distribution of PET tracers was performed to assess tumor biology [28] and tumor oxygenation status [29]. Metz et al. [28] reported weak to moderate correlations of PET with DCE-MRI by thresholding based on tracer uptake, although no significant correlations were found when the total tumor area was analyzed. Hansen et al. [29] reported a large variation in correlation coefficients between subjects. To the best of our knowledge, no study has compared the distribution and uptake of radiolabeled peptides with DCE-MRI-derived perfusion parameters in a neuroendocrine pancreatic tumor model in a voxel-wise manner.

In the present study, for all parameters it was found that low parameter values are associated with low peptide uptake. The highest correlation between DCE-MRI-derived parameters and peptide uptake was a Spearman’s correlation coefficient of 0.63. The parameters associated with the exchange between the blood vessels and extracellular extravascular space in the tumor (such as K^trans^, k_ep_, AUC60, TTP, wash-in and wash-out) showed higher correlations than the parameters primarily associated with the total amount of contrast delivered to the tumor (such as Smax and AUC). Furthermore, for the ‘exchange-related’ parameters a monotonic relation was observed with the peptide uptake (Figure 4). Regions with high ‘exchange-related’ parameter values showed higher peptide uptake than regions with low ‘exchange-related’ parameters. For the parameters associated with the total amount of contrast delivery, this association showed a distinct optimum: regions with higher AUC or Smax did not show a higher peptide uptake. This optimum could be explained by a saturation effect: a maximum level of peptide accumulation is reached when somatostatin receptors are saturated, after which further improvement of delivery will not result in a higher peptide uptake. As used in these studies, 10 µg of ^111^In-DTPA-octreotide will indeed partly saturate the tumor somatostatin receptors [30,31]. The ‘exchange-related’ parameters seem to predict peptide uptake better than the ‘contrast amount-related’ parameters. Consequently, fast and efficient diffusion through the vessel wall and into tissue is an important factor for peptide delivery. This information could offer a clue for improving peptide delivery. There are several methods available to enhance perfusion and/or permeability. For example, permeability inducing agents or hyperthermia, the latter enhances both perfusion and permeability [32].

The correlations found in this study are probably an underestimation, as several factors have limiting effects.

The contrast agent used in the MRI has different pharmacokinetic properties and thus is not an exact representation of peptide behavior after leaving the blood circulation. With DCE-MRI the circulation of contrast agent during the first 8 min after injection is measured, which provides information on influx into the tumor, vessel permeability, and diffusivity of the contrast agent in the tissue. SPECT is acquired over a period of 30 min, 1 h after administration of OctreoScan. The SPECT images provide information on peptide accumulation in tumor cells following circulation through the blood, diffusing through the vessel wall and into tissue, and subsequent binding and internalization. In other words, the peptide relies on tumor perfusion and permeability to reach the targeted cells, but final tumor uptake, as imaged with SPECT is also determined by peptide-receptor binding affinity and efficacy, and receptor expression levels. Therefore, a perfect correlation is not expected. Our data show that poor perfusion limits the amount of peptide able to reach the tumor, so that less peptide can bind to the receptor and internalize into the tumor cells. First and foremost the peptide needs to be delivered to the tumor; as such, the delivery of peptides can be adequately studied with a low molecular weight contrast agent.

Imperfection of registrations between SPECT and MRI could be another reason for lower correlations between SPECT and the DCE-MRI parameters. There is an inherent registration error between SPECT-CT and MRI: since CT and MRI image different tissue characteristics and have different distortions, tumor alignment will not be perfect between the modalities, implying that voxel-wise analysis will not be completely accurate. An alternative is to use larger voxels. Although this increases the overlap of voxels that are slightly misregistered, the use of less voxels also results in loss of information and homogenization of data.

Underestimation of the correlation can also be due to the ‘blooming effect’ that is inherent to SPECT imaging, together with the lower SPECT resolution. Regions with low perfusion/density/permeability, but close to a region with high SPECT uptake, will distort the correlation. This effect might also explain the large spread in median values shown in Figures 3 and 4.

Some tumor regions were identified with high DCE-MRI parameter values but were without peptide uptake as assessed by SPECT, as can be seen in the leftmost part of the tumor in Figure 2. Some of these regions were identified (based on SWI) as well vascularized connective tissue structures separating different lobes in the tumor; for other regions no visual clue was present. A homogeneous receptor expression for neuroendocrine tumors has been reported [16]. In a previous experiment with the CA20948 pancreatic tumor model, we also found a homogeneous receptor expression for vital tumor regions, as illustrated in Figure 1B. This high parameter value without peptide uptake can be explained by the dynamic perfusion of the tumor during the time frame of the different imaging sessions.

In the current study only a small number of animals were used. Since the aim of this study was to investigate whether uptake of radiolabeled peptide is correlated to tumor perfusion, vessel density and permeability, we feel that even with this limited set of CA 20948 tumors the correlations between SPECT and DCE-MRI parameters were adequately studied. In future studies, the effect of the tumor model on the correlation needs to be investigated.

In summary, for all tumors, areas with low peptide uptake also showed low values for all DCE-MRI-derived parameters, which could hamper therapeutic treatment with radiolabeled peptides in these areas. When all datasets were combined, a strong linear relation (R^2^ > 0.7) was found between radiolabeled peptide uptake imaged with SPECT, and either AUC60, TTP, wash-in, or wash-out. The average correlation coefficient between SPECT and DCE-MRI-derived parameters ranged from 0.52 to 0.56 for AUC60, TTP, wash-in, wash-out, K^trans^ and k_ep_. All these parameters are associated with the exchange between the blood and extracellular extravascular space in the tumor. For the parameters primarily associated with the total amount of contrast delivered to the tumor, such as Smax and AUC, the average correlation was relatively low (< 0.4). For these parameters a distinct optimum was observed: regions with higher Smax or AUC did not show higher peptide uptake. The ‘exchange-related’ parameters seemed to predict the peptide uptake better than the ‘contrast amount-related’ parameters. It can be concluded that fast and efficient diffusion over the vessel wall and into the tissue is an important factor for peptide delivery.

## Conclusion

By correlating radiopeptide uptake in a tumor, quantified by SPECT imaging, with DCE-MRI-derived parameters, we can conclude that the vascular characteristics of the tumor play an important role in the delivery of peptide and thus in targeting tumor cells. The ‘exchange-related’ parameters AUC60, TTP, wash-in, wash-out, K^trans^ and k_ep_ seemed to predict the peptide uptake better than the ‘contrast amount-related’ parameters AUC and Smax. Fast and efficient diffusion through the vessel wall and into tissue is an important factor for peptide delivery.

The application of DCE-MRI-derived parameters in assessing tumor perfusion, vessel density and permeability allows to study the effects of vascular characteristics on the treatment efficacy of PRRT. DCE-MRI can be used to understand treatment efficacy, and may even serve as a basis to make predictions on the delivery of radiopeptides in PRRT.
